# Noninfectious Granulomatous Lung Disease: Radiological Findings and Differential Diagnosis

**DOI:** 10.3390/jpm14020134

**Published:** 2024-01-23

**Authors:** Giulia Lassandro, Stefano Giusto Picchi, Antonio Corvino, Candida Massimo, Stefania Tamburrini, Laura Vanore, Giovanna Urraro, Giuseppe Russo, Francesco Lassandro

**Affiliations:** 1 Department of Radiology, Ospedale del Mare-ASL NA1 Centro, Via Enrico Russo 11, I-80147 Naples, Italy; giulia.lassandro@aslnapoli1centro.it (G.L.); stefanopicchi@libero.it (S.G.P.); stefanogiusto.picchi@aslnapoli1centro.it (S.T.); 2 Medical, Movement and Wellbeing Sciences Department, University of Naples “Parthenope”, Via Medina 40, I-80133 Naples, Italy; 3 Department of Radiology, Monaldi Hospital, A.O. Ospedali dei Colli, Via Leonardo Bianchi, I-80131 Naples, Italy; candida.massimo@ospedalideicolli.it; 4 Department of Radiology, Ospedale S. Anna e SS. Madonna della Neve, ASL NA3 Sud, Via Lenze, Boscotrecase, I-80042 Naples, Italy; laura.vanore@hotmail.it (L.V.); g.urraro@aslnapoli3sud.it (G.U.); f.lassandro@aslnapoli3sud.it (F.L.); 5 General Direction for Health Management, ASL Napoli 3 Sud, Via Marconi, Torre del Greco, I-80059 Naples, Italy; ariete_gr@libero.it

**Keywords:** chest imaging, granulomatous lung diseases (GLDs), radiology, high-resolution computed tomography (HRCT), differential diagnosis

## Abstract

Granulomatous lung diseases (GLDs) are a heterogeneous group of pathological entities that can have different clinical presentations and outcomes. Granulomas are histologically defined as focal aggregations of activated macrophages, Langerhans cells, and lymphocytes, and may form in the lungs when the immune system cannot eliminate a foreign antigen and attempts to barricade it. The diagnosis includes clinical evaluation, laboratory testing, and radiological imaging, which especially consists of high-resolution computed tomography. bronchoalveolar lavage, transbronchial needle aspiration or cryobiopsy, positron emission tomography, while genetic evaluation can improve the diagnostic accuracy. Differential diagnosis is challenging due to the numerous different imaging appearances with which GLDs may manifest. Indeed, GLDs include both infectious and noninfectious, and necrotizing and non-necrotizing granulomatous diseases and the imaging appearance of some GLDs may mimic malignancy, leading to confirmatory biopsy. The purposes of our review are to report the different noninfectious granulomatous entities and to show their various imaging features to help radiologists recognize them properly and make an accurate differential diagnosis.

## 1. Introduction

Granulomatous lung diseases (GLDs) are a heterogeneous group of pathological entities united by the presence of pulmonary granulomas. Granulomas are histologically defined as focal, organized inflammatory infiltrates of activated macrophages, Langerhans cells, and lymphocytes, and may form in the lungs when the immune system cannot eliminate a foreign antigen and attempts to barricade it [1,2]. Although granulomas are believed to form to encapsulate material or pathogens that cannot be eliminated otherwise, in some cases the exact causes for granuloma formation are still unclear [1,2].

GLDs can be primarily differentiated in infectious and noninfectious diseases. Infectious GLDs generally are caused by mycobacterial (both Mycobacterium tuberculosis and non-tuberculosis mycobacteria) and fungal (e.g., Histoplasma, Cryptococcus, Pneumocystis and Aspergillus) infections. Noninfectious GLDs include a variety of diseases, as inflammatory conditions (sarcoidosis), granuloma formation after environmental exposure (talc and berylliosis), vasculitis (granulomatosis with polyangiitis (GPA)), autoimmune diseases (rheumatoid nodule and lymphomatoid granulomatosis) and other types as pulmonary Langerhans cell histiocytosis [1,2,3]. A list of the noninfectious GLDs is reported in Table 1. Histologically, necrotizing and non-necrotizing granulomas have to be differentiated. Generally, necrotizing granulomas develop in association with an infectious cause [3].

The diagnosis includes clinical evaluation, laboratory testing, and radiological imaging, which especially consists of high-resolution computed tomography (HRCT). bronchoalveolar lavage (BAL), transbronchial needle aspiration or cryobiopsy, video-assisted thoracoscopic surgical (VATS) biopsy, and positron emission tomography (PET), while genetic evaluation can improve the diagnostic accuracy [3].

Direct evaluation of affected lung tissue is considered the gold standard in accessing GLDs [4]. Nevertheless, histological abnormality alone is rarely diagnostic for a specific granulomatous disorder, and the small size of tissue samples obtained by transbronchial needle aspiration or lung biopsy, together with high interobserver variability among pathologists, complicates the interpretation of histopathology [1]. In recent years, transbronchial cryobiopsy (TBCB) emerged as a procedure with higher diagnostic yield than transbronchial forceps biopsy (TBFB) for interstitial lung disease (ILD): in particular, an observational retrospective multicenter study including 276 patients with an ILD diagnosis who underwent TBCB or TBFB demonstrated that TBCB was safer and superior to forceps biopsy in the evaluation of both fibrotic and non-fibrotic ILDs [5].

In addition, as lung biopsies are not always readily available in the clinical setting and may be dangerous or non-suitable for the patient, a noninvasive technique such as HRCT is fundamental to diagnose the granulomatous inflammation of the lung [6]. HRCT allows medics to define the localization, number, and size of the granulomas, and to follow-up the lesions after treatment. Contrast-enhanced CT (CECT) and PET-CT might allow for the identification of additional sites with active lymphoproliferation that may be more easily accessible for biopsy (e.g., peripheral lymph nodes) [6]. Although radiology techniques (HRCT, CECT, and PET-CT) can adequately diagnose and follow-up the granulomatous lesions, differential diagnosis is challenging due to the numerous different imaging findings with which GLDs may manifest.

The purposes of our review are to report the different noninfectious granulomatous entities and to show their various imaging features to help radiologists recognize them properly and make an accurate differential diagnosis.

## 2. Sarcoidosis

### 2.1. Definition

Sarcoidosis is a multisystem granulomatous disease with heterogeneous clinical manifestations depending on the organs involved, but it primarily affects the lung and about 50% of patients are clinically asymptomatic [7].

Sarcoidosis has an incidence of 1–40 cases per 100,000 people per year and a prevalence of 0.2–64 cases per 100,000 people, with predilection for black people and for women [1,8].

The etiology of sarcoidosis is still unclear and numerous studies have shown that both genetic and environmental factors may contribute to pathogenesis [8,9]. Familial clustering in sarcoidosis has been reported in 4–17% of cases [10]. In addition, exposure to environmental factors such as insecticides, mold, inorganic particles, agricultural chemicals, and metal processing has been reported to increase the risk of sarcoidosis [11].

### 2.2. Micro-Macroscopic Features

Sarcoidosis is a cell-mediated immune response to unidentified antigens in genetically susceptible patients [8]. The histological hallmark of sarcoidosis is the presence of well-formed, compact, epithelioid, non-necrotizing granulomas with different degrees of lymphocytic inflammation [2,3,12]. Sarcoid granulomas contain epithelioid cells, macrophages, multinucleated giant cells, and CD4+ T cells in the center, while CD8+ T lymphocytes, B lymphocytes, and fibroblasts are seen at the periphery [13,14,15,16]. A frequent finding in sarcoidosis is the presence of intracytoplasmic inclusions, which are thought to represent products of macrophage metabolism that include asteroid bodies, Schaumann bodies, and calcium oxalate crystals [2].

In the lung, the sarcoidosis granulomas are typically distributed along the lymphatics in the peribronchovascular interstitium space (in both the perihilar regions and the lobular core) and, less frequently, in the interlobular septa and subpleural interstitial space [17]: this typical distribution explains why transbronchial biopsies are so effective in detecting sarcoid granulomas [2].

### 2.3. Clinical Findings

As mentioned above, clinical presentation of sarcoidosis may be variable, with nonspecific respiratory symptoms such as cough, dyspnea, and chest pain, or more typical symptoms such as ocular abnormalities, ocular, lacrimal and salivary gland involvement, cutaneous lesions, and peripheral lymphadenopathy [12,13,18].

Usually there is a long delay in diagnosis because about 50% of patients are asymptomatic despite the presence of abnormalities on chest radiographs [8,12].

Patients may also present specific acute clinical symptoms of systemic sarcoidosis, known as Löfgren syndrome, which manifests with lymphadenopathy, fever, erythema nodosum, polyarticular arthritis, and occasionally uveitis and parotitis. Another less common sarcoidosis presentation is the Heerfordt syndrome, with symptoms including fever, parotid enlargement, facial palsy, and anterior uveitis [19,20].

### 2.4. Diagnosis

A multidisciplinary approach with laboratory tests, clinical history, physical examination, and radiological and pathological evaluation is fundamental in the case of clinical suspicion of sarcoidosis [21]. Laboratory tests include markers such as angiotensin-converting enzyme, serum interleukin-2 receptor, neopterin, chitotriosidase, lysozyme, plasma N-terminal pro-brain natriuretic peptide, and troponin T, indicative of the disease activity [8,12]. 

Chest X-ray is the first-line imaging technique in pulmonary sarcoidosis, due to its sensitivity, as radiological lung abnormalities can be found in more than 90% of patients with pulmonary sarcoidosis, but HRCT is far more specific and allows for a comprehensive evaluation [8,11,22].

**Figure 1:**
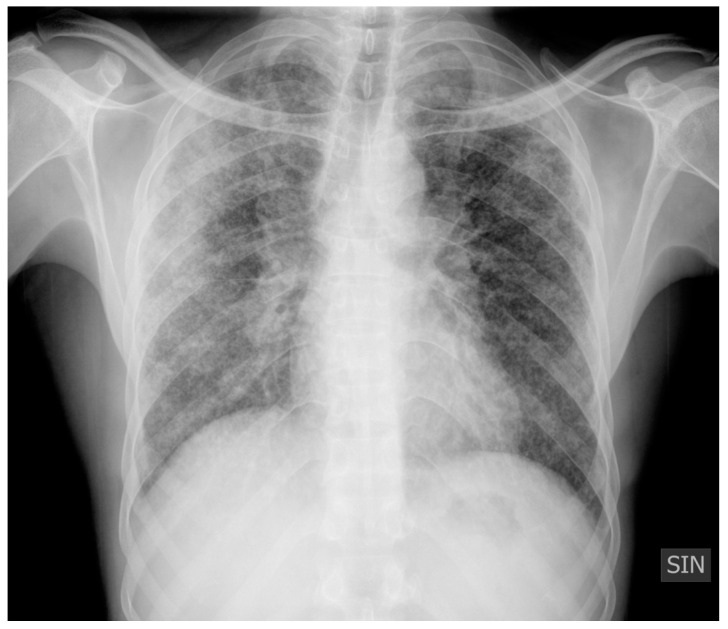
Chest X-ray, AP, showing a range of pulmonary parenchymal abnormalities: multiple micronodules with a peribronchovascular distribution, reticulonodular opacities, right lung consolidation, and symmetric hilar lymphadenopathy. According to Scadding classification, the X-ray in the Figure can be classified as stage II (thoracic adenopathy and pulmonary infiltrates), as in 25–65% of patients.

Chest X-ray may show a range of lung parenchymal alterations including micro- and macro-nodules often with a peribronchovascular distribution, reticulonodular opacities, and signs of pulmonary fibrosis (Figure 1). Mediastinal lymphadenopathy is commonly seen in the right paratracheal and aorto-pulmonary window nodal stations regions; symmetric hilar lymphadenopathy is also a typical finding in lung sarcoidosis (Figure 1) [23,24,25].

In 1961, Scadding reported a radiographic staging system for sarcoidosis: stage I consists of thoracic adenopathy (25–65% of patients), stage II consists of thoracic adenopathy and pulmonary infiltrates (25–65% of patients), stage III consists of pulmonary infiltrates alone (10–15% of patients), and stage IV consists of pulmonary fibrosis (5% of patients) [26].

While chest X-ray frequently shows the mediastinal lymphadenopathy, it is less sensitive and specific then HRCT in evaluating the lung parenchymal abnormalities. In fact, further evaluations are frequently performed with HRCT of the chest [7]. HRCT allows clinicians to obtain thin-slice chest images, post-processed in a high spatial frequency reconstruction algorithm, leading to optimal lung detail for assessment of pulmonary parenchymal abnormalities.

**Figure 2:**
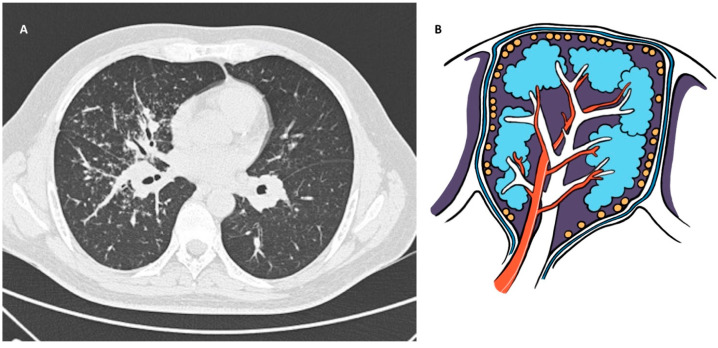
(**A**): Chest HRCT, axial planes, shows well-defined micronodules, prominent in the right lung, with a perilymphatic distribution along the bronchovascular bundles, interlobular septa, interlobar fissures, and subpleural regions; there are also larger nodules in the middle lung zones and symmetric hilar lymphadenopathy. (**B**): Original drawing representing the perilymphatic disposition of parenchymal micronodules.

Typical HRCT findings seen in pulmonary sarcoidosis are well-defined 2–5 mm micronodules with a perilymphatic distribution along the bronchovascular bundles, interlobular septa, interlobar fissures, and subpleural regions (Figure 2). Less common findings are larger nodules occurring more commonly in the upper lung lobes or masses with surrounding satellite nodules (“galaxy” sign), ground-glass opacities (GGO) or peribronchovascular consolidation, air trapping, and fibrosis that may be present in 20–25% of patients and tends to be more central with predominance in the upper and middle lobes [7,8,24,27].

Sarcoidosis typically manifests with symmetric mediastinal lymph nodes, perilymphatic micronodules, and fibrotic changes, but sometimes it can be associated with a wide range of imaging features such as unilateral hilar or mediastinal adenopathy, which can mimic lymphoma, metastatic lymph nodes, tuberculosis or other granulomatous diseases [25,28,29,30].

Differential diagnoses include bronchiolitis, lymphangitic carcinomatosis, hypersensitivity pneumonitis (HP), pulmonary Langerhans cell histiocytosis (PLCH), and interstitial lung diseases (ILDs) such as usual interstitial pneumonia (UIP), cryptogenic organizing pneumonia, and nonspecific interstitial pneumonia (NSIP) [8].

The differential diagnosis in the case of fibrotic changes in pulmonary sarcoidosis is based on the location of the parenchymal abnormalities that are typically seen in the upper lung lobes, in contrast to the UIP that are located in the lower lobes with peripherical distribution.

Sarcoidosis may have a similar clinical and radiological picture to sub-acute HP, but the exposure history, the presence of specific IgG antibodies against antigen, and lymphocytosis on BAL support a diagnosis of HP, while pathological examination in sarcoidosis typically reveals well-formed non-caseating granulomas along the bronchovascular bundle, without inflammatory cell infiltrate [1,31]. Air trapping is an HRCT finding both in pulmonary sarcoidosis and in HP. Sarcoidosis can be also differentiated from chronic HP by the different distribution of the micronodules (perilymphatic/subpleural/along fissures in sarcoidosis versus centrilobular in HP) and by the lack of mosaic perfusion in sarcoidosis [1,2].

The typical perilymphatic distribution of micronodules is the main feature to differentiate sarcoidosis from PLCH, which also predominate at the upper lobes but have centrilobular location. In addition, mediastinal and hilar adenopathy are absent in PLCH [25,32].

BAL is one of the minimally invasive investigational tools for the differential diagnosis of diffuse parenchymal lung diseases [33,34]. The typical findings of BAL in pulmonary sarcoidosis include a normal or mildly elevated total cell count with lymphocytosis(CD4+/CD8+ ratio > 3.5 is high specific), a normal percentage of eosinophils and neutrophils, and an absence of plasma cells and foamy alveolar macrophages; active sarcoidosis tends to show higher lymphocyte counts than inactive sarcoidosis [35].

The diagnosis of sarcoidosis is made by the combination of clinical features, radiological findings, pathological results and biopsy; the histological diagnosis of sarcoidosis was made significantly more often by TBLC than by TBLF [36]. 

## 3. ANCA-Associated GLDs

### 3.1. Granulomatosis with Polyangiitis (Wegener Granulomatosis)

#### 3.1.1. Definition

GPA, also known as Wegener granulomatosis, is defined as systemic vasculitis that predominantly affects small vessels, usually without eosinophilia. Eosinophils may be absent or present in small numbers, but only rarely are numerous [2,37].

With eosinophilic granulomatosis with polyangiitis (EGPA, formerly known as Churg–Strauss syndrome), GPA is now included in the group termed antineutrophil cytoplasmic antibody (ANCA)-associated vasculitis [38].

#### 3.1.2. Micro-Macroscopic Features

Histologically, GPA consists of necrotizing granulomas associated with necrotizing vasculitis [37]. The granulomas are suppurative, usually irregular in contour, with basophilic appearance due to the presence of neutrophils and nuclear debris. The suppurative necrosis is surrounded by palisading histiocytes, acute and chronic inflammation, and granulation tissue. Multinucleated giant cells, when present, are distinctive but not pathognomonic for GPA. They stand out at low magnification because of the presence of multiple, closely packed, hyperchromatic nuclei [39]. In contrast, compact non-necrotizing, sarcoidosis-like granulomas are exceptional in GPA.

#### 3.1.3. Clinical Findings

GPA generally manifests with a classic triad of upper and lower respiratory tract symptoms, and glomerulonephritis with hematuria [40]. 

The incidence of thoracic involvement in GPA is up to 75% [41], and the lung lesions may be multiple or rarely solitary [42]. Renal involvement may be absent at presentation [2,42].

#### 3.1.4. Diagnosis

One of the key findings of GPA is the presence of ANCA in up to 90% of systemic forms and in about 50% of localized forms. Notably, PR3-ANCAs are highly specific to GPA and have high value in the diagnosis [43,44].

As the incidence of thoracic involvement in GPA is up to 75%, HRCT is the mainstay in evaluating patients with GPA who have suspected thoracic involvement [41].

**Figure 3:**
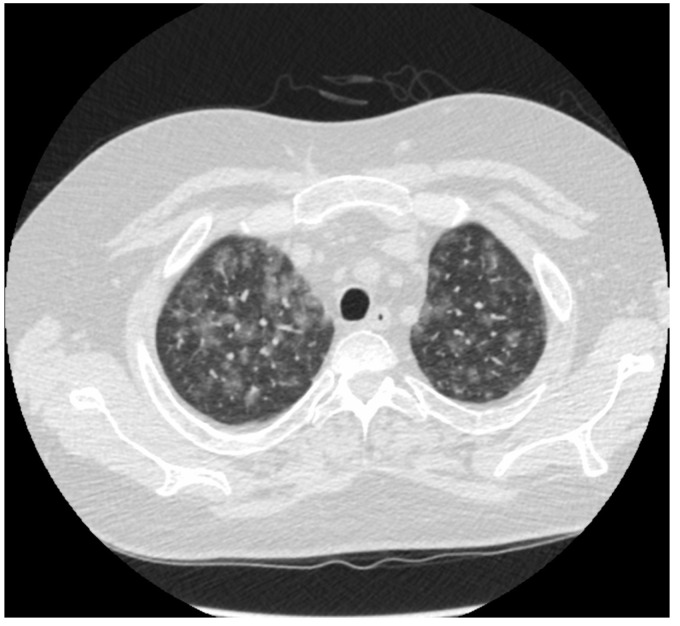
Chest HRCT, axial planes, showing numerous bronchiolocentric GGO, with symmetric distribution.

**Figure 4:**
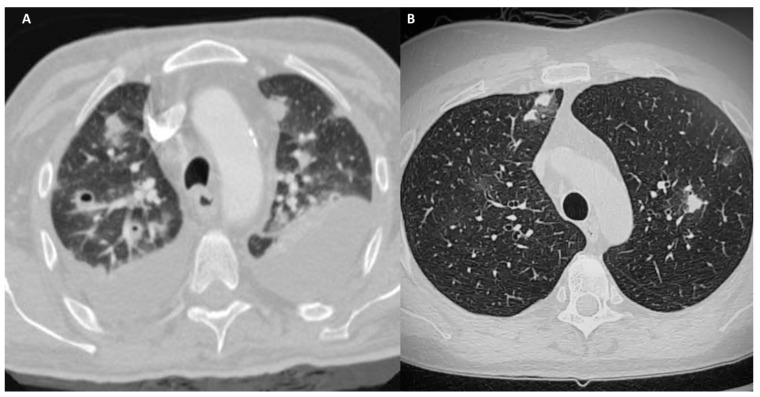
(**A**): Chest HRCT, axial planes, patient affected by GPA: CT scans shows bronchiolocentric nodules, several cavitated, surrounded by GGO halo as a result of alveolar inflammation or diffuse alveolar hemorrhage secondary to necrotizing vasculitis. Bilateral pleural effusion can be noted. (**B**): Chest HRCT, axial planes, patient affected by EGPA: CT scans shows bronchiolocentric nodules with perilesional GGO halo as a result of alveolar inflammation/hemorrhage secondary to necrotizing vasculitis. In these two figures, it is shown that the HRCT differential diagnosis between GPA and EGPA can only be the presence of central cavitation of the lesions. Pleural effusion is more frequent in EGPA, seen in up to 50% of cases, and may be secondary to eosinophilic cardiomyopathy or eosinophilic pleuritis. In this case, it is present in GPA and not in EGPA HRCT.

The most frequent finding in GPA are nodules, GGO (Figure 3), or nodules/masses with or without central cavitation (Figure 4A), typically bronchiolocentric. Nodules or masses can be also surrounded by GGO halo as a result of alveolar inflammation or diffuse alveolar hemorrhage secondary to necrotizing capillaritis. Differential diagnoses include bronchiolitis, abscesses, metastasis, and other GLDs as eosinophilic granulomatosis with polyangiitis (EGPA) and PLCH. The differential diagnoses with EGPA and PLCH will be discussed later in the respective paragraphs for a better understanding of radiological diagnosis.

Centrilobular tree-in-bud micronodules can be found in up to 10% of patients, related to bronchiolar wall involvement or to blood products retained in the distal airways. Wall thickening of segmental and subsegmental bronchi can be found in 70% of patients with GPA, while more centrally located airway involvement is far less common [40]. Notably in these cases, the differential diagnosis with bronchiolitis can be challenging.

Most of both lung and airway disease findings improve after treatment [40].

Although GPA usually affects small vessels, large-vessel involvement is also possible, more frequently in conjunction with pulmonary and small-vessel findings. Pleural effusions can occur in GPA, while mediastinal lymphadenopathy is uncommon.

### 3.2. Eosinophilic Granulomatosis with Polyangiitis (Churg–Strauss Syndrome)

#### 3.2.1. Definition

EGPA, formerly known as Churg–Strauss syndrome, is a rare vasculitis that demonstrates necrotizing granulomas at histopathologic evaluation. GPA is included in the group of ANCA-associated small-vessel vasculitis, in which EGPA is the least common [38].

#### 3.2.2. Micro-Macroscopic Features

In EGPA granulomas are well formed, with central necrosis rich in eosinophils [45] and small-vessels necrotizing vasculitis, which is also rich in multinucleated giant cells and eosinophils.

The disease involves small vessels and distal bronchioles, the surrounding alveolar spaces are filled with eosinophils, and it may cause eosinophilic pneumonia. In fact, the classic histologic picture in the lung consists of necrotizing granulomas, necrotizing vasculitis, and eosinophilic pneumonia, but this triad is rarely found [46,47]. Therefore, the diagnosis is rarely based of histologic findings alone.

#### 3.2.3. Clinical Findings

The most common clinical findings are asthma, pulmonary infiltrates, neuropathy (mono or poly), and peripheral eosinophilia, with extravascular eosinophils seen in biopsy specimens.

Since clinical diagnostic criteria vary widely, definitive diagnosis requires ANCA testing [46,47].

#### 3.2.4. Diagnosis

Chest X-ray abnormalities are nonspecific in EGPA, due to the presence of bilateral, multifocal, non-segmental, and typically peripheral consolidations [48].

HRCT is the best noninvasive technique in the characterization of the lung parenchymal findings of EGPA. The more common HRCT findings are bronchiolocentric nodules (both small or big ones) or GGO or consolidation, bronchial wall thickening, bronchial dilatation, interlobular septal thickening, and mosaic attenuation.

Small nodules correspond to eosinophilic bronchiolitis and peribronchiolar vasculitis, while bronchial wall thickening is usually from airway wall eosinophilic and lymphocytic infiltration [49]. As in case of GPA, nodules can be surrounded by GGO halo as a result of alveolar inflammation or diffuse alveolar hemorrhage secondary to necrotizing capillaritis.

Pleural effusion can be noted in up to 50% of patients and can be related to eosinophilic cardiomyopathy or pleuritis. Mediastinal lymph nodes are not frequent and manifest in less than 25% of cases [40,50]. Patients are more prone to thromboembolic phenomena due to eosinophilia, especially pulmonary embolism [51].

The main entities to consider in the histologic differential diagnosis are infection (fungal as Aspergillus or Coccidioides or parasites such as Dirofilaria), eosinophilic pneumonia, and Wegener granulomatosis.

The differential diagnosis with infectious diseases and eosinophilic pneumonia is clinically very challenging because of their very similar clinical features [46], but histological necrotizing vasculitis and ANCA positivity allow a non-radiological differential diagnosis.

With GPA the differential diagnosis is far more difficult, both clinically and radiologically. GPA frequently shows destructive upper respiratory tract lesions and ANCA positivity, but not the asthma or peripheral blood eosinophilia that is common in EGPA.

HRCT allows clinicians to differentiate EGPA from GPA not for the lesions location as in both of these diseases the abnormalities are bronchiolocentric, but for the cavitation that is frequent in GPA and very uncommon in EGPA (Figure 4) [7,52].

## 4. Rheumatoid Nodule

### 4.1. Definition

Rheumatoid nodules consist of necrotizing granulomas commonly associated with rheumatoid arthritis (about 20% of patients), and rarely with inflammatory bowel disease. These nodules can be single or multiple, with subcutaneous and lung locations [1].

### 4.2. Micro-Macroscopic Features

The typical histologic appearance is that of a necrotizing granuloma with abundant central necrosis and a rim of palisading epithelioid histiocytes. There is often basophilic karyorrhectic debris at the interface between the necrosis and the granulomatous rim. There may be associated vasculitis but necrotizing vasculitis is not found [45].

### 4.3. Clinical Findings

Rheumatoid nodules arise in patients affected by rheumatoid arthritis. Typically, these nodules are asymptomatic, but rupture can occur, resulting in infection, pleural effusion, or bronchopleural fistulas [53]. The nodules typically regress with treatment of rheumatoid arthritis.

### 4.4. Diagnosis

Rheumatoid nodules predominately distribute in subpleural sites, with a size that varies from 1 to 10 mm, and often cavitate. Therefore, the HRCT differential diagnosis with necrotizing vasculitis GPA and EGPA is challenging, notably with GPA for the presence of cavitated lesions.

The diagnosis of rheumatoid nodules is essentially one of exclusion. In fact, the diagnosis of rheumatoid nodules should be considered when necrotizing granulomas are encountered in the lung of a patient affected by rheumatoid arthritis. The clinical context is fundamental, since most rheumatoid nodules are multiple and subpleural and occur in seropositive patients with active joint disease [54].

## 5. Secondary to Substances Inhalation GLDs

### 5.1. Hypersensitivity Pneumonitis

#### 5.1.1. Definition

HP, also known as extrinsic allergic alveolitis, consists of a syndrome caused by repeated exposure to and inhalation of a variety of environmental antigenic particles or chemical agents, resulting in noncaseating granulomas formation in the lungs [1,7,55]. More common subtypes of HP include hot tub lung, bird fancier’s lung, and farmer’s lung [7].

The individual differences in susceptibility to HP suggests a genetic link likely through major histocompatibility complex (MHC) class II, namely, HLA-DR and DQ [31]. Cigarette smoking seems to protect from developing clinically significant HP likely due to nicotine inhibiting macrophage activation and lymphocyte proliferation [31].

HP has conventionally been classified into acute, subacute, and chronic subtypes based on the disease duration at the time of onset. Nevertheless, according to updated knowledge and revised diagnostic criteria, in 2020 the American Thoracic Society (ATS) recommended to classify HP in fibrotic and non-fibrotic form, differentiating HP forms on the presence/absence of radiological and histopathological fibrosis [31,56].

#### 5.1.2. Micro-Macroscopic Features

In HP, the lung parenchymal inflammation results in a combination of type-III and type-IV hypersensitivity reactions. After initial sensitization, the offending or chemical agent triggers an immune-complex-mediated (type III) hypersensitivity reaction; in fact, in acute HP, high titers of specific IgG antibodies can be detected in serum. Repeated exposure to the antigen leads to a delayed (type IV) hypersensitivity reaction with activation of CD8 cytotoxic T cells, macrophage activation, and granuloma formation [31].

In the non-fibrotic HP form, pathologic findings are poorly formed non-caseating granulomas with bronchiolocentric location, inflammatory lymphocytes infiltrates, and foci of organizing pneumonia consisting of small airways filled with fibroblastic plugs (Masson bodies) [31].

In the fibrotic form, an exaggerated T-cell-mediated immune response can be seen, leading to collagen deposition and fibrosis. In fact, in the fibrotic HP form, pulmonary fibrosis occurs, and sparse poorly formed non-caseating granulomas and chronic inflammation are seen around the bronchioles [31].

#### 5.1.3. Clinical Finding

Clinical presentation of HP is influenced by the type and the amount of the antigen, the intensity and frequency of exposure, and the host immune response [1,55]. The onset of symptoms may be acute (days to weeks) or insidious (month to years) with gradually worsening of symptoms [55]. 

Acute non-fibrotic HP symptoms at presentation are fever, fatigue, cough, and dyspnea. Symptoms usually start after a few hours of heavy exposure to nonspecific antigens and resolve in 1–2 days of avoiding exposure, while prolonged exposure to the sensitized antigen results in the fibrotic form, with shortness of breath and weight loss [31,55].

At physical examination, inspiratory crackles or squeaks that reflect small airway involvement can be heard on pulmonary examination and in fibrotic stage HP, crackles may be more prominent [55].

#### 5.1.4. Diagnosis

Diagnosis of HP can be challenging and requires a combination of detailed history, laboratory tests, pulmonary function tests (PFTs), imaging findings, and pathological examination.

Identification of the offending agent is crucial in the diagnosing and treatment of HP [31]. Spirometry often reveals a restriction pattern with decreased diffusing capacity on PFTs [55].

Chest X-ray and HRCT are usually the first-line imaging techniques in the assessment of HP [1], although chest X-ray has low sensitivity and is almost always normal [57]. Abnormal radiographic findings may include, in the non-fibrotic form, numerous small (<5 mm) patchy or diffuse airspace opacities, typically sparing apices and bases throughout both lungs; in fibrotic form, there may be a reticular pattern predominant in the upper lobes that can also show volume loss [31].

**Figure 5:**
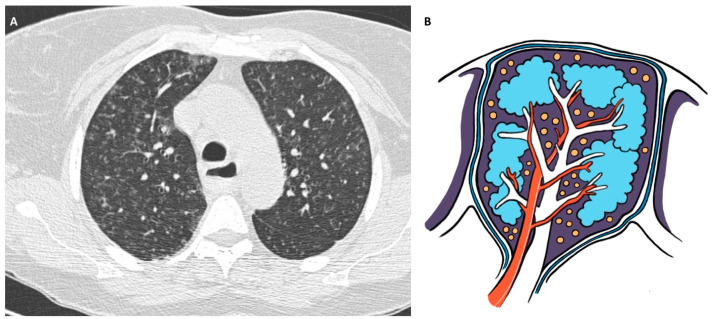
(**A**): Chest HRCT, axial planes, shows HP in acute stage with diffuse centrilobular poorly defined small nodules and ground-glass attenuation with prevalence to upper lobes. (**B**): Original drawing representing the centrilobular disposition of parenchymal micronodules.

HRCT is the main imaging technique in the HP diagnosis [55]. In non-fibrotic form, the typical HRCT findings are upper and middle lobe patchy or diffuse GGO and centrilobular poorly defined small nodules (<5 mm), referred to as the “headcheese sign” (Figure 5A,B); mosaic attenuation (air-trapping) is also observed, notably on expiratory phase HRCT scan, as a sign of small airway obstruction due to bronchiolitis [1,31,56]. The accuracy of HRCT diagnosis can be about 92% [31]. In the fibrotic form, the prominent findings on HRCT are the signs of lung fibrosis (interlobular septal thickening, lobar volume loss, linear/reticular opacities, traction bronchiectasis, and honeycombing) combined with GGO and centrilobular small nodules. Thin-walled cystic changes and mediastinal lymphadenopathy can also be seen in chronic HP [31,56].

The differential diagnoses for non-fibrotic HP include infections of the respiratory tract, metal fume fever, and organic dust toxic; clinical history, and physical and radiologic examination should help differentiate the conditions [31]. The primary differential diagnosis for non-fibrotic HP is sarcoidosis, which has a similar clinical and radiologic picture; exposure history, the presence of IgG antibodies against specific antigens, and lymphocytosis on BAL support a diagnosis of HP, while in sarcoidosis, pathological examination typically reveals well-formed non-caseating granulomas along the bronchovascular bundle, without inflammatory cell infiltrates [1,31]. 

The differential diagnoses for fibrotic HP is ILDs such as UIP, idiopathic pulmonary fibrosis (IPF), and NSIP; reticulation and honeycombing can make it difficult to make a distinction from UIP or NSIP, but the relative sparing of bases and the air trapping favors HP over UIP or NSIP [58,59]. Fibrotic HP should also be differentiated from sarcoidosis by the different distribution of the micronodules (centrilobular in HP versus perilymphatic/subpleural/along fissures in sarcoidosis) and by the lack of mosaic perfusion in sarcoidosis [1,2].

Flexible bronchoscopy with BAL is a highly sensitive method to detect HP: a remarkable increment of lymphocytes (usually >50%) and low CD4:CD8 ratio are characteristics for HP. If a definitive diagnosis cannot be reached after a multidisciplinary evaluation, then a lung biopsy should be performed [31].

### 5.2. Aspiration Pneumonia

#### 5.2.1. Definition

Pulmonary aspiration of a variety of substances, including oropharyngeal bacteria, foreign bodies, and gastric contents can lead to an acute necrotizing bronchopneumonia or to chronic granulomatous inflammation of the airways and lungs, known as “aspiration pneumonia”. Aspiration of gastric contents is the most common cause of this type of pneumonia and can be a significant cause of morbidity and mortality [60,61].

#### 5.2.2. Micro- and Macroscopic Features

Histologically, aspiration pneumonia is typically characterized by acute inflammation and bronchiolocentric necrosis with remnants of aspirated material surrounded by multinucleated giant cells [62,63].

#### 5.2.3. Clinical Findings

Aspiration pneumonia is frequently associated with predisposing factors such as hiatus hernia, structural abnormalities of the pharynx and esophagus, features supportive of reflux or neurologic disorders such as a stroke in clinical history or dementia, or also emergency surgical procedures [60,64].

#### 5.2.4. Diagnosis

Radiological findings can vary depending on the duration, the material that was aspirated, the amount of material aspirated, and whether the aspiration has caused an underlying pneumonia, which may or may not be present [61].

Simple aspiration is typically associated with centrilobular nodules, some with a tree-in-bud appearance, due to the distribution of the aspirated material in the distal airways, especially in lower zone, which is gravity-dependent [65]. The differential diagnoses include bronchiolitis and other GLDs secondary to substances inhalation such as talc granulomatosis.

**Figure 6:**
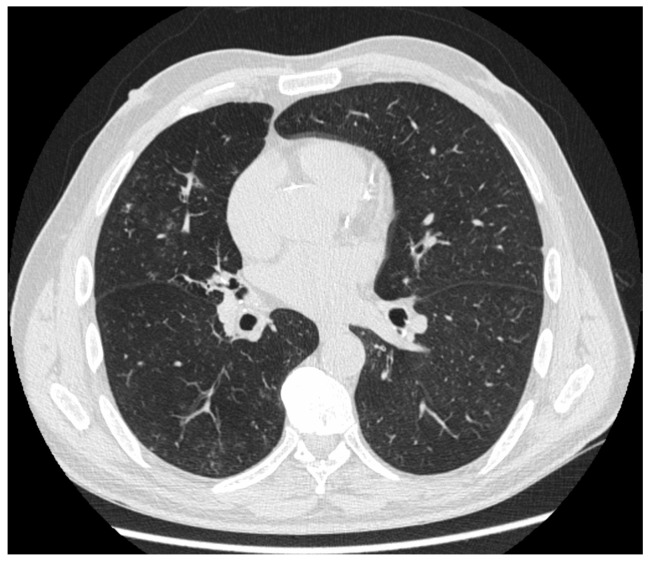
Chest HRCT, axial planes, patient affected by chronic aspiration pneumonia. CT scan shows centrilobular micronodules and GGO due to the surrounding parenchyma inflammation, and occlusive aspiration of the medium lobe bronchus, with relative pulmonary atelectasis.

Chronic aspiration pneumonia may be associated with granulomas formation, and centrilobular GGO or foci of consolidation due to the surrounding parenchyma inflammation. When aspiration is occlusive, atelectasis may be present (Figure 6). Bronchial wall thickening and bronchiectasis/bronchiolectasis often can be seen, particularly in patients with chronic or repeated aspiration [7,66].

**Figure 7:**
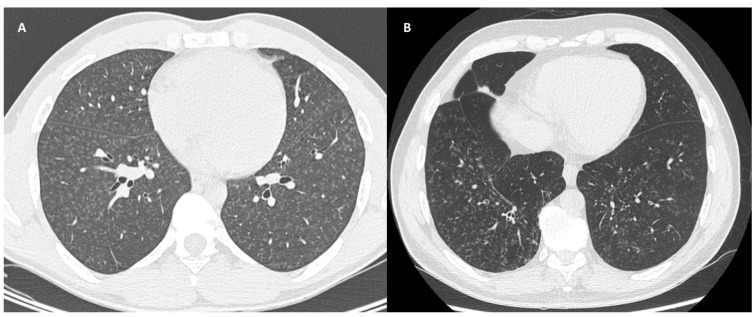
Chest HRCT, axial planes, patient affected by talc granulomatosis (**A**) and patient affected by aspiration pneumonia (**B**). In both (**A**,**B**), CT shows numerous millimetric micronodules in a centrilobular pattern. The differential diagnosis between aspiration pneumonia and talc granulomatosis, when occlusive bronchus involvement and pulmonary atelectasis are not present, can be very challenging and impossible without a detailed clinical history, bronchoscopy, and BAL.

While each feature on its own can be nonspecific, the most common radiographic pattern is that of bronchopneumonia with scattered air-space opacities, manifesting as diffuse, small (1–3 mm) nodular areas of increased opacity representing the bronchiolar distribution of the aspirated material (Figure 7B) [67].

The differential diagnosis Ides infections, GPA, and talc granulomatosis. In GPA, the presence of bronchocentric granulomas, often cavitated, and acute inflammation may make it challenging to differentiate from aspiration, but bronchoscopy and BAL can demonstrate aspirated particulate material in the case of aspiration pneumonia [2].

The key to differentiate talc granulomatosis is the location of the granulomas: in aspiration pneumonia they occur in peribronchiolar parenchyma, while in talc granulomatosis, they are in the alveolar septa [2].

### 5.3. Talc Granulomatosis

#### 5.3.1. Definition

Talc (hydrated magnesium silicate) is frequently used in industrial manufacturing and as an excipient in oral medications. Talc deposition in the lungs can occur in case of either inhalation (i.e., pneumoconiosis) or intravenous administration. In this second modality, generally it is present as aqueous suspensions of crushed oral medications such as methadone, methylphenidate, tripelennamine, and pentazocine [68,69].

#### 5.3.2. Micro-Macroscopic Features

Talc is identified histologically within the granulomas and since it is the most common material found and, in most cases, it comprises the bulk of the material, the term “talc granulomatosis” is widely used [69].

#### 5.3.3. Clinical Findings

When talc is injected, it results in innumerable tiny talc particles trapped in the pulmonary capillary bed and in foreign-body-type granulomatous response around these vessels [70].

These patients experience progressive dyspnea and pulmonary function decline. Obstruction of blood flow by the foreign material and the associated granulomatous response may lead to thrombosis, vascular dilatation, and pulmonary hypertensive changes [70].

#### 5.3.4. Diagnosis

A chest radiograph is generally insensitive [7] or may show nonspecific abnormalities in the case of advanced clinical pictures.

In the case of intravenous talc administration, HRCT typically shows numerous millimetric micronodules in a centrilobular pattern. This is the specific anatomic deposition of these particles.

In case of talc inhalation, HRCT can show conglomerate high attenuation masses, like calcium conglomerates [71,72]. The high attenuation is uncommon, but not impossible, in the case of talc particles injection [71].

The differential diagnosis includes aspiration pneumonia, without occlusive bronchus involvement and pulmonary atelectasis (Figure 7). In most cases, talc granulomatosis nodules may show a higher attenuation of pulmonary micronodules, resembling calcium, and this can differentiate it from aspiration pneumonia [71,72]. Nevertheless, a detailed clinical history, bronchoscopy, and BAL are fundamental to differentiate these GLDs.

### 5.4. Berylliosis

#### 5.4.1. Definition

Berylliosis is an occupational GLD, resulting in relevant exposure to beryllium, usually in manufacturing industries such as the electronics, automotive, aerospace, and nuclear industries [73,74].

In fact, berylliosis only rarely occurs in the general population, most frequently in persons living close to a beryllium processing plant or with family members that have been exposed to the contaminated clothes of beryllium workers [75].

The disease is dose-dependent, and higher exposure puts patients at higher risk for berylliosis [76], but only 1–5% of exposed persons develop the disease.

#### 5.4.2. Micro-Macroscopic Features

Berylliosis is characterized by a granulomatous reaction in the lung to inhaled beryllium. Granulomas are non-caseating and poorly formed and are associated with interstitial inflammation with evidence of mononuclear cells [73]. The interstitial inflammation is very similar to hypersensitivity pneumonitis.

#### 5.4.3. Clinical Findings

Because it is an occupational disease, patients are most commonly adult men of working age, typically adults with a reported mean age at diagnosis of 44 years [74].

Symptoms are nonspecific, similar to asthmatic ones such as dry cough and shortness of breath, or less frequently with fever, fatigue, night sweats, and weight loss [73,77].

#### 5.4.4. Diagnosis

Berylliosis is phenotypically indistinguishable from sarcoidosis, as clinical, radiological, and histopathological findings mimic sarcoidosis. In fact, more than 6% of patients diagnosed with sarcoidosis might suffer from berylliosis [74,78].

HRCT shows pulmonary micronodules with perilymphatic/subpleural/along fissures distribution and bilateral hilar or mediastinal lymph node involvement [2], indistinguishable from sarcoidosis without a history of exposure to beryllium. 

Therefore, the diagnosis can be made by a positive beryllium lymphocyte proliferation test [73].

## 6. Lymphocytic Interstitial Pneumonia

### 6.1. Definition

Lymphocytic interstitial pneumonia (or pneumonitis, LIP) is a rare form of ILD, a benign lymphoproliferative disorder consisting of infiltration of lung tissue with lymphocytes and plasma cells [79].

LIP can occur at any age with a mean age of 52–56 years, with a female predilection because women are more likely to develop autoimmune diseases [79,80]. LIP occurs more frequently in people affected by autoimmune disorders or immunodeficiency, such as Sjogren’s syndrome, systemic lupus erythematosus, autoimmune thyroid disease, human immunodeficiency virus (HIV) infection, and multicentric Castleman’s disease [81].

### 6.2. Micro-Macroscopic Features

LIP is considered a benign lymphoproliferative disorder histologically characterized by diffuse interstitial and alveolar infiltration with polyclonal lymphocytes and plasma cells [82]. In this disease, the inflammatory infiltration of the interstitium by reactive T and B lymphocytes, plasma cells, and histiocytes can be seen, with consequential inflammation of lung parenchyma and bronchi [79,81,83]. The main pathologic feature of LIP is dense interstitial lymphocytic infiltrates, which expand and widen the interlobular and alveolar septa [79,83].

In about 80% of patients, polyclonal or IgM monoclonal gammopathy is found [80].

### 6.3. Clinical Findings

LIP clinical presentation has gradual onset and symptom duration usually ranging from 2 months to 12 years before medical evaluation; when symptoms are present, the most common are dyspnea and non-productive cough with approximately 6 months duration [79,84]. Less common symptoms are systemic ones, such as fever, night sweat, arthralgia, weight loss, salivary glands hypertrophy and respiratory failure, cyanosis and clubbing in the end-stage disease [84].

Approximately 5% of cases may transform into lymphoma [85].

### 6.4. Diagnosis

Radiological findings of LIP may vary widely [81]. Chest X-ray shows nonspecific findings, the most frequent are classically bilateral lower zone reticular or reticulonodular opacity, while less common findings include a nodular pattern, GGO, and air-space consolidation [86].

**Figure 8:**
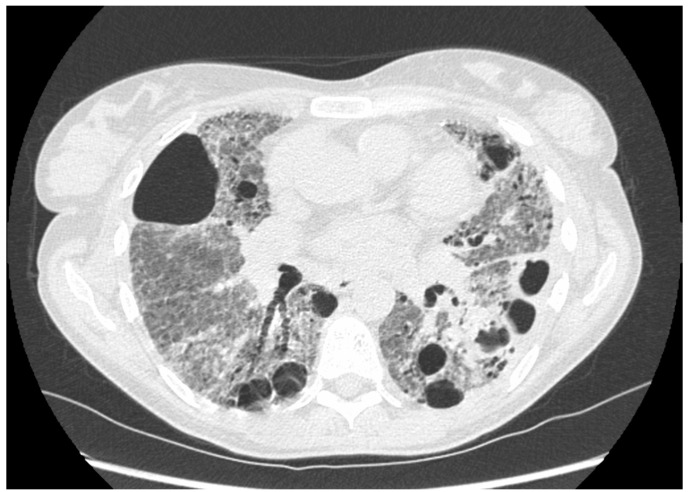
Chest HRCT, axial planes: CT scan shows GGO, subpleural nodules or consolidations, bronchovascular bundles thickening, interlobular septal thickening, thin-walled cystic airspaces with perivascular distribution, and architectural distortion.

HRCT allows for establishing the extent of disease, identifying pleural or mediastinal/hilar involvement, and for differentiating LIP from other diffuse pulmonary diseases [83,87]. The classic HRCT findings are GGO, poorly defined central lobular and subpleural nodules, thickening of the bronchovascular bundles, interlobular septal thickening, thin-walled cystic airspaces (notably with perivascular distribution), and lymph node enlargement (Figure 8); less common findings are large nodules, emphysema, airspace consolidation, bronchiectasis, architectural distortion, honeycombing, and pleural thickening [79,88].

The differential diagnoses of LIP include Pneumocystis pneumonia, lymphangioleiomyomatosis (LAM), PLCH, primary malignant lymphoma of the lung, and NSIP [89]. 

Pneumocystis pneumonia is the most frequent opportunistic infection in patients with HIV, with cysts variable in appearance and with upper zone predominance, while in LIP there is a predominance in lower lung lobes [90].

The presence of many thin-walled, round, bilateral lung lobes cysts, with normal intermediate lung parenchyma, in a young woman is virtually pathognomonic of LAM, while in LIP, cystic airspaces have generally perivascular distribution [89].

Both LIP and PLCH are characterized by centrilobular nodules and cystic airspaces, mainly located in the upper and middle lung fields, but in case of LIP, interlobular thickening and hilar lymph nodes are also common [89].

In LIP, lung lesions are diffuse, while in primary malignant lung lymphoma there is typically a localized lesion; these findings may allow a radiological differentiation of these diseases [88].

In NSIP, there are diffuse and uniform distributions of pulmonary lesions, with some alveolar walls uninvolved and without structural remodeling; the extent of inflammatory cell infiltration is less than that of LIP [88].

BAL is also a valuable indicator in the diagnosis of the disease: the increase in lymphocytes, CD3 cells, and polyclonal CD20 cells is generally indicative of LIP [83,88]. However, the final diagnosis of LIP depends on lung biopsy, pathologically consisting of diffuse lymphocytic infiltration in the pulmonary interstitium. Immunohistochemical studies are needed to determine the polyclonal nature of lymphocytes and to differentiate LIP from lymphoma [88].

## 7. Pulmonary Langerhans Cell Histiocytosis

### 7.1. Definition

PLCH, synonymous with eosinophilic granuloma, is a rare GLD within Langerhans and other inflammatory cells accumulate in bronchiolar structures, leading to nodular inflammatory lesions formation [91]. PLCH represents less than 5% of all ILDs of unknown etiology [32].

It is a rare disease without any predilection for individuals of either sex, but primarily affects white patients [1]. PLCH usually occurs in young adult, in >90% of cases smokers or ex-smokers, therefore, smoking appears to be one of the important predisposing factors [1], with a peak incidence between the ages of 20 and 40 years [92].

### 7.2. Micro-Macroscopic Features

PLCH is characterized by prominent peribronchial inflammatory abnormalities [31], suggesting small airways injury by an inhaled irritant such as cigarette smoke. The pathogenesis of PLCH probably involves cigarette-smoke-induced epithelial cells and macrophage recruitment and accumulation around small airways, interstitium, and distal air spaces [91]. These cells produce cytokines and chemokines that promote the recruitment, retention, and activation of Langerhans cells CD1a-positive monoclonal. In addition, cigarette smoke action can directly activate Langerhans cells. These Langerhans cells CD1a-positive monoclonal may inappropriately recognize auto-antigens in the lungs and activate adaptive T-cell responses, leading to airways injury and granulomas formation. Chronic inflammation and cytokine production may activate local fibroblasts and promote airway-centered fibrosis that destroys and remodels surrounding lung tissue and promote distal bronchiolar dilatation with cystic formation [91].

### 7.3. Clinical Findings

Clinical presentation of PLCH is usually nonspecific, and symptoms may include nonproductive cough and dyspnea in about 65% of patients, or less common symptoms such as fever, chest pain, fatigue, and weight loss. Acute presentation with a spontaneous pneumothorax is described in about 15–20% of cases [91,92]. Hemoptysis occurs rarely and justifies the search for possible complications such as infectious bronchitis, lung cancer, or aspergillus colonization of a cystic cavity [32]. Nevertheless, in about 25–30% of cases, patients affected by PLCH are asymptomatic and the disease can be incidentally found on chest X-ray PLCH [93,94].

### 7.4. Diagnosis

PLCH is generally suspected on clinical history and on chest X-ray, but it can be confirmed by HRCT, bronchoscopy with biopsy, and BAL [95].

Initial PFTs can be normal or show a mild, predominantly obstructive or mixed pattern, while diffusion capacity is the most frequently compromised aspect at disease onset [32,93,96]. Routine laboratory tests are nonspecific.

PLCH can be strongly suspected on radiological imaging techniques [32]. PLCH has variable appearance depending on the stage of the disease, ranging from small peribronchiolar nodular opacities to multiple irregularly shaped cysts [95]. Chest X-ray is almost always abnormal, although the findings may be subtle [91,97]. The earliest changes are diffuse, bilateral, symmetrical reticulonodular lesions prominent in the mid and upper zones, while in advanced disease, cystic lesions and fibrotic changes are more dominant [97]. Pleural effusion and significantly enlarged mediastinal lymph nodes are rarely observed [92].

**Figure 9:**
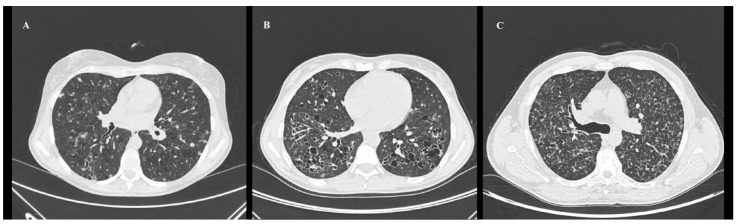
Chest HRCT, axial planes, shows PLCH in early, advanced, and end stage (**A**–**C**). (**A**): In early stage disease, there are nodular lesions corresponding to “florid” granulomas, characterized by brochiolocentric and ill-defined micronodules surrounded by ground-glass opacification secondary to inflammatory interstitial infiltration, nodules with irregular margins and centrilobular distribution, and cavitary nodules with thick walls that later become cysts. (**B**): In advanced stage disease, cystic lesions usually predominate on nodules: cysts appear round and of small dimension (<10 mm), but in advanced disease they are typically larger and of different shapes with thick-walled cysts (>2 mm thick) that progressively transform into thin-walled cysts (<2 mm thick). (**C**): In end-stage disease, there is a fibrocystic pattern that maintains the typical upper- and middle-lung zone predominance.

HRCT is the best technique in identifying both the reticulonodular opacities, cysts, and fibrotic changes with the typical upper- and middle-lung zone predominance (Figure 9A–C) [95,98]. In early stages, florid granulomas are represented by brochiolocentric, ill-defined micronodules or nodules, eventually surrounded by GGO secondary to inflammatory interstitial infiltration (Figure 9A) [32,96]. Generally, nodules are stellate or with irregular margins, number from a few to innumerable, have a diameter from 1 mm to 10 mm (typically 1–5 mm), centrilobular distribution; less frequently, they may also be peribronchial or peribronchiolar and may be cavitary nodules with thick walls later becoming cysts [32,95,96]. In advanced stages, cystic lesions predominate on nodules because the inflammatory activity is decreased: in advance disease, cysts are typically larger with thick-walled cysts (>2 mm thick) that progressively transform into thin-walled cysts (<2 mm thick) (Figure 9B) [32]. End-stage disease consists of a fibro-cystic pattern that maintains the typical upper- and middle-lung zone predominance (Figure 9C) [32].

Differential diagnosis depends on whether the nodular or cystic change is the dominant feature. In early stages, differential diagnoses include lung metastasis, miliary tuberculosis, silicosis, sarcoidosis, GPA, and respiratory bronchiolitis (RB)-ILD [32]. Later in the disease, principal differential diagnoses include UIP, centrilobular emphysema, LAM, and LIP [32,99,100,101]. Typical PCLH micronodules centrilobular location allows clinicians to differentiate it from sarcoidosis and silicosis, which also predominate at the upper regions but are characterized by perilymphatic distribution of nodules. In addition, in PLCH, mediastinal and hilar lymph nodes are absent [25,32]. Early PLCH can have the same features as RB-ILD, presenting as bronchiolocentric ill-defined micronodules generally associated with mild centrilobular emphysema [32]. Differential diagnosis with GPA can be challenging due to the bronchiolocentric distribution of micronodules, often cavitated [102,103,104,105].

In advanced disease, PLCH is easy to differentiate from UIP due to the typical basal alterations, including the layered arrangement of cysts, intralobular interstitial thickening, and traction bronchiectasis [32]. Cyst morphology can help to differentiate LAM from PLCH, with more regular shape in LAM and with bizarre ones in advanced PLCH; in LAM, cysts are also randomly distributed without sparing the costophrenic angles and parenchymal nodules are less common [32].

In adults, BAL is highly indicative of PLCH when the quantity of CD1a-positive cells is >5% of total cells [91,93]. Definitive diagnosis can be achieved with lung biopsy, generally performed by VATS, consisting of identification of Langerhans cell proliferation with respiratory bronchiolar infiltration [91,93,95].

## 8. Conclusions

In this review, the aim was to discuss the broad clinical, pathological, and radiological spectrum of noninfectious GLDs.

HRCT can be a fundamental tool in noninvasive GLD confirmation, in the recognition and differential diagnosis in the different GLDs, especially in clinically doubtful cases. Since, as mentioned above, GLDs are not rarely associated with nonspecific symptoms or atypical clinical presentations, HRCT represents a noninvasive technique that allows for a GLD to be recognized even when it does not fit the clinical suspicions and to make a differential diagnosis between different GLDs.

Nevertheless, clinical signs and symptoms and laboratory tests are essential in the diagnosis of GLDs and their specific recognition. In fact, GLDs diagnosis requires a multidisciplinary approach of pulmonologists, radiologists, pathologists, immunologists, and rheumatologists. A combined diagnostic approach taking clinical presentation, laboratory workup, imaging techniques, histologic findings, and genetics results into consideration can, therefore, help to shape the way to personalized diagnosis and treatment.

The purposes of our review are to report the different noninfectious GLDs and to show their various imaging features to help radiologists recognize them properly and make an accurate differential diagnosis.

## Figures and Tables

**Table 1:** A list of non-infectious GLDs grouped according to etiology.

Non-Infectious GLDs	
Inflammatory	Sarcoidosis
ANCA-associated vasculitis	-Granulomatosis with polyangiitis -Eosinophilic granulomatosis with polyangiitis
Rheumatoid arthritis manifestations	Rheumatoid nodule
Secondary to substances inhalation	-Hypersensitivity pneumonitis -Aspiration pneumonia -Talc granulomatosis -Berylliosis
Lymphoproliferative	Lymphocytic interstitial pneumonitis
Pulmonary Langerhans cell histiocytosis	

## Data Availability

Not applicable.
